# Genetic polymorphism of merozoite surface protein-1 and merozoite surface protein-2 in *Plasmodium falciparum *isolates from Brazzaville, Republic of Congo

**DOI:** 10.1186/1475-2875-10-276

**Published:** 2011-09-22

**Authors:** Pembe Issamou Mayengue, Mathieu Ndounga, Freddy Vladimir Malonga, Michel Bitemo, Francine Ntoumi

**Affiliations:** 1 Fondation Congolaise pour la Recherche Médicale, BP 2672 Brazzaville, Republic of Congo; 2 Faculty of Health Sciences, University Marien Ngouabi, BP 2672, Brazzaville, Republic of Congo; 3 Laboratoire de Pharmacologie, Centre d'Etudes sur les Ressources Végétales, BP 1249 Brazzaville, Republic of Congo; 4 Centre de Recherche et d'Etude en Sciences Sociales et Humaines, BP 2019 Brazzaville, Republic of Congo; 5 Institute for Tropical Medicine, University of Tübingen, 72074 Tübingen, Germany

## Abstract

**Background:**

The characterization of malaria parasite populations circulating in an area is part of site characterization, as a basis for evaluating the impact of malaria interventions on genetic diversity, parasite species, and multiplicity of infection. The present study was aimed at analysing genetic diversity of *Plasmodium falciparum *merozoite surface proteins 1 and 2 (MSP-1 and MSP-2) and to determine the multiplicity of infection in clinical isolates collected from children living in the Southern district of Brazzaville in the Republic of Congo.

**Methods:**

A total of 125 isolates from patients with uncomplicated malaria attending Terinkyo and Madibou health centres were collected between January and June 2005 while evaluating the therapeutic efficacy of amodiaquine-artesunate combination. DNA was extracted and *msp-1 *and *msp-2 *genes were genotyped using allele-specific nested-PCR.

**Results:**

Out of 468 distinct fragments detected, 15 *msp-1 *and 20 *msp-2 *genotypes were identified. For the *msp-1 *gene, K1 family was the predominant allelic type carried alone or in association with RO33 and Mad20 types, whereas the 3D7 family was the most prevalent in the *msp-2 *gene. Overall, the mean multiplicity of infection was 2.2. Out of 125 samples, 104 (83%) harboured more than one parasite genotype. There was no statistical significant difference in the multiplicity of infection by either sex or age of patients. However, a statistically significant correlation was found between parasite densities and the number of genotypes.

**Conclusion:**

Polymorphism in *P. falciparum *clinical isolates from Brazzaville was high and mainly of multiple clones. The basis for the positive association between parasite densities and multiplicity of infection is discussed.

## Background

Malaria, a disease mostly caused by *Plasmodium falciparum*, is a major public health problem. The global burden is estimated at 225 million malaria cases every year resulting into 781,000 deaths [1], sub-Saharan Africa being the most affected region. In the Republic of Congo, like in many other sub-Saharan African endemic countries, malaria vulnerable groups are children and pregnant women [2].

Data collected from 2000 to 2003 by the University Teaching Hospital of Brazzaville during studies on chloroquine resistance showed that 22% of children deaths at pediatric health facilities were due to malaria [3]. Recent studies on anti-malarial drug resistance in Brazzaville have confirmed high level chloroquine resistance and the inefficacy of sulphadoxine-pyrimethamine and amodiaquine either singly or in combination for the treatment of uncomplicated malaria [4-6]. Consequently, in February 2006, the Republic of Congo changed its anti-malarial drug policy for treating uncomplicated malaria to artesunate-amodiaquine combination, as the first-line drug, and artemether-lumefantrine the second-line thus meeting the recommendation of the World Health Organization.

Despite policy change, there is still need to collect more data on the epidemiology of malaria in the country to facilitate the development and implementation of other malaria control interventions such as the search for alternative drugs, vaccine trials and indoor residual spraying. In this regards, characterization of malaria parasite populations circulating in an area is part of site characterization before any intervention, as a basis for evaluating the impact of the intervention on genetic diversity, parasite species, and multiplicity of infection (MOI) in the human or vector host. One of the limitations to the development of an effective malaria vaccine against *P. falciparum *is the extensive genetic diversity in parasite populations limiting the efficacy of acquired protective immunity to malaria [7]. MOI and demonstration of presence of parasite in blood are among the most useful indicators of clinical disease [8-11]. In the human host, MOI is defined as the mean number of detected *P. falciparum *genotypes per infected individual.

Asexual blood stages antigens, such as merozoite surface protein-1 (MSP-1) and merozoite surface protein-2 (MSP-2) are considered prime candidates for the development of malaria vaccine and are also suitable markers for the identification of genetically distinct *P. falciparum *parasite sub-populations [12]. Polymorphism in *msp-1 *and *msp-2 *has been reported in several parts of world including in Central Africa [13-15]. However, there is limited data on the genetic diversity among *P. falciparum *populations from the Republic of Congo.

Molecular studies to identify *P. falciparum *genes mutations associated with anti-malarial drugs resistance, such as those found in *P. falciparum *chloroquine resistance transporter, dihydrofolatereductase, dihydropteroate synthase have been conducted in the Republic of Congo since 2003, before introducing artemisinin-based combination treatment (ACT) in 2006 [5,16,17]. Base on the literature, only one publication has reported the genetic diversity of *P. falciparum *infections and MOI in a group of children with uncomplicated malaria in Brazzaville [5]. Following massive deployment of insecticide treated nets and free treatment with ACT for malaria in the country, it is important to evaluate the genetic profile of malaria strains before and during these effective interventions.

The present characterized allelic polymorphism of *msp-1 *and *msp-2 *and determined the multiplicity of infection in clinical *P. falciparum *isolates collected from children living in the southern district of Brazzaville, the capital of the Republic of Congo. The Ministry of Public Health of the Republic of Congo approved the study ethically and scientifically.

## Methods

### Study area

The study materials were collected from patients attending Centre de Santé Intégré (CSI) in Terinkyo and Madibou hospitals, located in the southern part of Brazzaville. This urban area has stable and highly endemic malaria with perennial transmission [18] with an entomological inoculation rate (EIR) of 22.5 infective bites/person/year was reported [19]. Malaria infection is primarily due to *P. falciparum*.

### Study population and blood samples collection

A total of 125 *P. falciparum *infected blood used in this study were collected from children and adults presenting to the two health centres and enrolled into the study for evaluating the therapeutic efficacy of artesunate-amodiaquine combination (Dr. Ndounga, unpublished data) from February to June 2005. The inclusion criteria of participants were: auxiliary temperature ≥ 37.5°C measured with an electronic thermometer, malaria parasite (*P. falciparum*) in the thick blood smears (≥ 800 parasites/μL), no symptoms of severe malaria or febrile conditions caused by other diseases. Informed consent was obtained from adult patients and from parents/guardians for the children. Additionally, at inclusion into the study and before treatment, three drops of blood from each patient were blotted on to 3 MM Whatman filter paper, dried and stored in individual sealed envelopes until used for DNA extraction.

### Extraction of parasite DNA

Genomic DNA was extracted from blood samples collected on filter paper using QiaAmp DNA mini kit (Qiagen, Hilden, Germany) according to the manufacturer's instruction. Then, the DNA was recovered in 100 μl of elution buffer from the kit. All parasites DNA extracted were stored at - 20°C until use.

### Allelic typing of *P. falciparum msp-1 *and *msp-2 *genes

All samples were genotyped for *P. falciparum *using the nested polymerase chain reactions (PCRs) technique. The highly polymorphic loci, *msp-1 *block 2 and *msp-2 *central region were used as markers for this genotyping as described previously [11-13]. The initial amplifications were followed by individual nested PCR reactions using specific primers for K1, MAD20, and RO33 allelic families of *msp-1*, and for FC27 and 3D7 allelic families of *msp-2*. Allelic specific positive controls and DNA free negative controls were included in each set of reaction. Five microliters of each of the PCR products were loaded on 2% agarose gel, stained with the Syber Green, separated by electrophoresis and visualized under UV trans-illumination. Individual alleles were identified by fragment length and by the corresponding allele-specific primers used and the size of the PCR products were estimated using a 100 bp DNA ladder marker (Invitrogen, Karlsruhe, Germany). The size polymorphism in each allelic family was analysed; assuming that one band represented one amplified PCR fragment derived from a single copy of *P. falciparum msp-1 *or *msp-2 *genes. Alleles in each family were considered the same if fragment size were within 20 bp interval. The minimum number of genotypes per isolate was estimated to be the highest number of fragments identified for either *msp-1 *or *msp-2*.

### Malaria species genotyping

To confirm that all study participants were infected with only *P. falciparum*, all samples were subjected to *Plasmodium *species genotyping as described elsewhere [20]. At the second PCR round, only *Plasmodium malariae *and *Plasmodium ovale *species were investigated; *P. falciparum *being already done by *msp-1 *and *msp-2 *genotyping. The PCR products were loaded on 2% agarose gels and visualized under UV trans-illumination. The DNA positive controls for *P. malariae *and *P. ovale *as well as DNA free negative controls were included in each set of reaction.

### Data and statistical analysis

The *msp-1 *and *msp-2 *allele frequency was calculated as the proportion of allele found for the allelic family out of the alleles detected in isolates. The multiplicity of infection was defined as the minimum number of *P. falciparum *genotypes per infected subject and estimated by dividing the number of amplified PCR fragments reflecting parasite genotypes by the number of positive samples. The chi-square test was used to compare proportions. Spearman's rank correlation coefficients were calculated to assess association between MOI and geometric mean parasite densities and age. Statistical significance was defined as *P *values < 0.05.

## Results

### Characteristics of patients and parasitaemia

A total of 125 patients with clinical symptoms of malaria and microscopically confirmed *P. falciparum *infection were enrolled in the study. Sixty-seven and 48 were children aged between seven months to five years of age, and between six to 15 years of age, respectively; ten patients were more than 16 years old. Sixty-eight patients were males and 57 were females.

The geometric mean parasite density was 62,170 parasites/μl of blood with the range of 830-914,000 parasites/μl (Table 1).

**Table 1:** Characteristics of individuals with uncomplicated *P.falciparum *malaria from Brazzaville, Republic of Congo

Characteristics of patients	**No**.	Values
No. of Male/Female	125	68/57
Mean age (years)	125	8 (0.7-54)
Mean parasite density (parasites/μl)	125	62,170 (830-914,000)
Multiplicity of *P. falciparum *infection	125	2.2
Polyclonal infections (%)	104	83

### *Plasmodium *species

Isolates from 125 patients were PCR-typed for speciation and all were confirmed to contain *P*. *falciparum*. Of these, two isolates were mixed infections of *P. falciparum/P. malariae *and *P. falciparum/P. ovale*.

### Allelic polymorphism of *P. falciparum *(*msp-1 *and *msp-2*)

**Figure 1:**
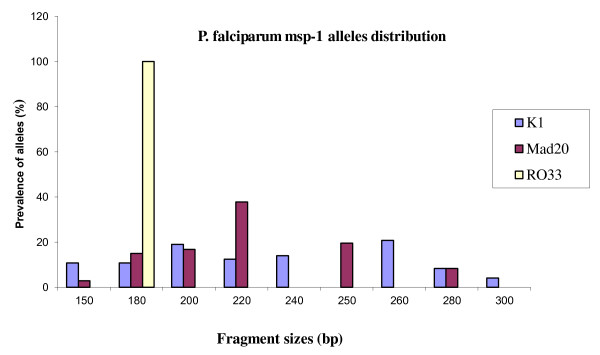
**Prevalence of *P. falciparum *msp-1 alleles in Congolese clinical isolates**. Alleles of the msp-1 gene were classified by fragment length (in base pairs) resulting from PCR amplifications with allele specific primers.

The parasite DNA from all 125 *P. falciparum *isolates were analysed for *msp-1 *and *msp-2 *genes, whereby only one isolate did not amplify for *msp-1 *gene. The efficiency of *msp-1 *and *msp-2 *genes amplification reactions with family-specific primers was 100% and 95%, respectively. A total of 468 distinct fragments were detected, out of which, 15 *msp-1 *and 20 *msp-2 *different alleles were identified. Within the *msp-1 *gene (Figure 1), eight and six alleles belonged to K1 (48% of overall detected *msp-1 *alleles) and Mad20 (29%) families, respectively. The RO33 (23%) family did not show any polymorphism, with only one variant (180 bp) detected. The K1 family was the predominant allelic type carried alone (23.4%) or in mixed infection with RO33 (17%) and Mad20 (20%) types as well as when the tree types are observed in the same isolates (Table 2).

**Table 2:** Distribution of msp1 and msp2 detected allelic families and corresponding mean parasite densities in Congolese *P.falciparum *isolates

	Mean Parasite Densities		
MSP1		MSP2	
	FC27	3D7	**FC27+3D7**

K1	65273	58899	**59110**
Mad20	89818	76886	**75476**
RO33	60116	57269	**61454**
**K1+Mad20**	**73789**	**63524**	**61976**
**RO33+K1**	**62561**	**55788**	**57151**
**RO33+Mad20**	**82289**	**70746**	**70738**
**RO33+K1+Mad20**	**73789**	**64104**	**62465**

In bold are presented multiclonal infections.

**Figure 2:**
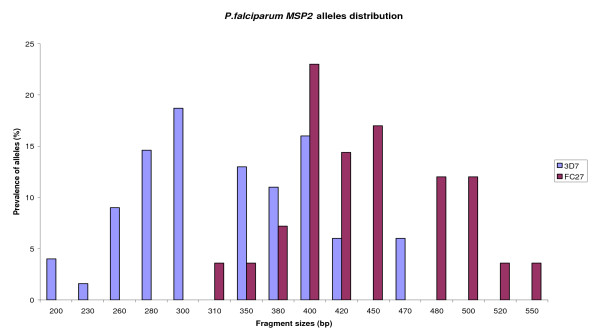
**Prevalence of different *P. falciparum *msp-2 alleles in Congolese clinical isolates**.

The *msp-2 *gene yielded 10 alleles for each family (Figure 2). The 3D7 family was more prevalent with 57% of overall detected *msp-2 *alleles compared to 38% of FC27. Forty-four (35.5%) of the isolates carried both *msp-2 *allelic types. Ten genotypes (5%) could not be assigned to any family.

### Relationship between multiplicity of infection, parasite densities, age and gender

**Figure 3:**
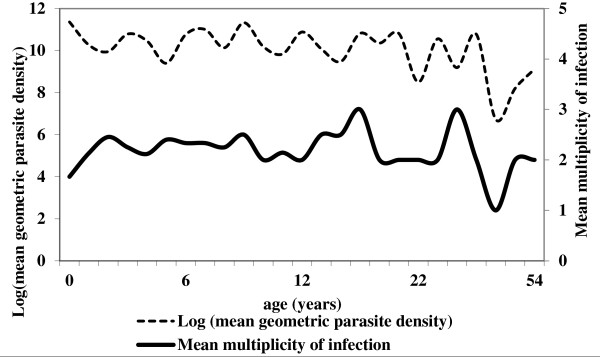
**Relation between multiplicity of *P. falciparum *infection as well as mean geometric density with age**.

**Figure 4:**
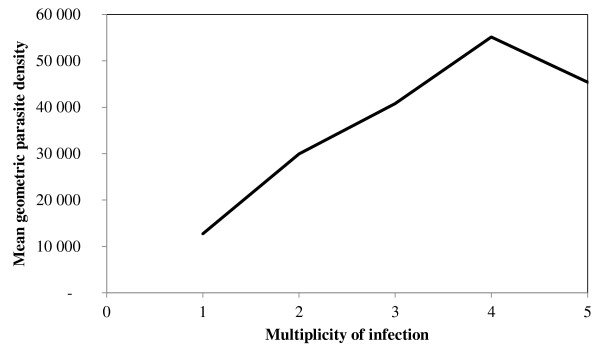
**Relation between mean geometric parasite density and the multiplicity of *P. falciparum *infection**.

Overall, the mean multiplicity of infection was 2.2. Of 125 samples, 104 (83%) harboured more than one parasite genotype. When considering *msp-1 *and *msp-2 *genes separately, the overall multiplicity of infection was 2 and 1.7 respectively, while eighty-nine (72%) and 67 (54%) of isolates carried more than one parasite genotype respectively. As presented on the Tables 2 and 3 the type of combination of multiple *msp-1 *or *msp-2 *alleles did not show any impact on the parasite density. Moreover, no statistical significant difference was observed in the multiplicity of infection and parasite density according to the age (Figure 3) and gender. However, a significant correlation was observed between mean parasite densities and the number of alleles (Spearman rank coefficient for *msp-1 *= 0.1784; p = 0.0474, and Spearman rank coefficient for *msp-2 *= 0.1834; p = 0.0406). The correlation was higher when *msp-1 *and *msp-2 *alleles are taken together (Spearman rank coefficient = 0.2508; p = 0.0048), since the number of genotypes per isolate was calculated as the highest number of fragments for either *msp-1 *or *msp-2 *(Figure 4).

**Table 3:** Profile of multiple infections according to the parasite densities (*P.falciparum *parasites/μl of blood)

Multiplicity of Infection	Mean Parasite Densities
**1**	30,490
**2**	64,979
**3**	70,216
**4**	90,632
**5**	45,390
**Total**	62,170

## Discussion

The level of antigenic diversity of *P. falciparum *populations in an area is likely to affect acquisition of immunity to malaria [21]. Therefore, the understanding of the genetic structure of parasite population is necessary for planning of malaria control interventions.

Allelic specific *P. falciparum msp-1 *and *msp-2 *genotyping has shown that malaria parasite population in Brazzaville is highly diverse. However, the number of alleles may have been underestimated due to the limit of the technique. Indeed, fragments within the length interval of less than 20 bp could not clearly be distinguished. Thus, a minimum of fifteen alleles of *msp-1 *were observed, in which, K1 allelic family was predominant, consistent with the previous study in the same locality and others studies in the central Africa sub-region [5,13,22,23]. Although Mad20 family was diverse, the distribution of Mad20 alleles was almost equal to single detected RO33 allele. Regarding the *msp-2 *locus, alleles belonging to 3D7 family were mostly detected, both in mono-infection and mixed infection with FC27 alleles. This finding differs from those of the previous study [5], where RO33 and K1 families were similarly distributed, and FC27 alleles more predominant. The difference may either be due to a small sample size in the first study or the existence of a true difference in the two study areas. Despite the fact that both studies were done during the same time of the year, the study population for the first study conducted in 2003 lived in rural Southern areas, just after the armed conflicts in the Republic of Congo. Therefore, malaria parasite profiles found in this investigation may have not reflected the true parasite picture compared to the current study. Repeated studies are required to characterize and confirm the real genetic diversity of parasite populations in this area. Limited polymorphism in RO33 family is similar to findings in Senegal [24], Brazil [25] and in Iran [26]. Three and 4 RO33 alleles were found in Gabon [23] and Uganda [27] respectively. The current parasite profile with the predominance of K1 and 3D7 was also observed in isolates from patients with uncomplicated *P*. *falciparum *malaria in Gabon [22,23].

Multiple genotypes of *P. falciparum *infections were detected in 83% of Congolese isolates with an overall mean multiplicity of infection of 2.2 (with 2 and 1.7 in *msp-1 *and *msp-2 *respectively). This corroborates reports from other areas of Central Africa with intense malaria transmission [22,28]. In this study, the number of parasite genotypes carried by subjects with symptomatic infections was not influenced by age as shown elsewhere [29]. Irrespective of age, the sensitivity of the technique may not allow for the detection of minor genotypes when the parasite density of disease-inducing strain is high.

However, in contrast with the first report in Brazzaville [5], *P. falciparum *infection in this symptomatic population shows a significant correlation between parasite density and the number of parasite genotype. This suggests that symptomatic *P*. *falciparum *infection in this population is associated with the presence and growth of recently inoculated parasite strain(s) to which individuals have either not previously been exposed to or have not fully developed immunity [13,30]. This observation in Congolese population is in agreement with the study conducted in Bangui [29] and in Mozambique [31]. The *msp-1 *and *msp-2 *genes appeared polymorphic, and both taken together, the relation between parasite density and multiplicity of infection become more pronounced. This finding supposed that in area with high transmission where individuals are exposed to several different clones, disease-inducing strains may have similar growing capacity leading to the increase of their parasite densities. Consequently, with the technique used, the high density may increase the probability to distinguish more clones in the complex infections.

Despite the lack of recent entomological data from Brazzaville, the latest estimation being in 1985 [18], the genetic diversity and multiplicity of infection found in the present study confirm intense malaria transmission in the area. Most likely, fluctuations in alleles were related to variations of the allele ratios in peripheral blood, as observed over several months in the absence of malaria transmission [32]. In this study, blood samples were collected during the rainy season of January to June, when malaria transmission is very intense. All year round studies covering dry and rainy seasons are needed to get the real picture of genetic profiles in this area including a sense on seasonal variations.

With regard to malaria speciation, two isolates had mixed infections of *P. falciparum *+ *P. malariae *and *P. falciparum *+ *P. ovale*, confirming high sensitivity of molecular tools in detecting sub-microscopic infection.

## Conclusion

The description of malaria parasite population remains a priority malaria research area in the Republic of Congo for a better understanding and evaluation of on-going malaria control interventions. This study was conducted before the implementation of the new policy for the treatment of uncomplicated malaria using ACT. Assessment of the impact of ACT on malaria parasite population and characterization of mutations in *P. falciparum *genes associated with anti-malarial drug resistance would be useful developments.

## Conflict of interest

The authors declare that they have no competing interests.

## Authors' contributions

PIM and FVM conducted molecular genetic analysis. The four authors participated in the design of the study, analysis and interpretation of the results, as well as in preparing the manuscript. All authors read and approved the final manuscript.
